# Effect of Ti Content on Microstructure and Properties of Laser-Clad Fe-Cr-Ni-Nb-Ti Multi-Principal Element Alloy Coatings

**DOI:** 10.3390/ma18173985

**Published:** 2025-08-26

**Authors:** Jie Yang, Zhe Zhang, Xiaoming Chen, Yidong Wu, Hui Liu, Zhao Dong, Xidong Hui

**Affiliations:** 1 China Yangtze Power Co., Ltd., Yichang 443002, China; 2 State Key Laboratory for Advanced Metals and Materials, University of Science and Technology Beijing, Beijing 100083, China; 3 Key Laboratory of Surface Engineering of Equipment for Hydraulic Engineering of Zhejiang Province, Standard and Quality Control Research Institute, Ministry of Water Resources, Hangzhou 310024, China

**Keywords:** laser-clad, multi-principal element alloy, friction wear, cavitation erosion

## Abstract

Multi-principal element alloy (MPEA) Fe_66.5−x_Cr_24_Ni_8_Nb_1.5_Ti_x_ coatings were fabricated via laser cladding on Q235 steel substrates to enhance their surface performance. This study revealed that the Ti content within these MPEAs directly influences the properties of the resulting coatings. The experimental results demonstrated that increasing the Ti content in the Fe_66.5−x_Cr_24_Ni_8_Nb_1.5_Ti_x_ MPEA promoted the formation of a BCC phase, significantly enhancing the coatings’ mechanical properties. Specifically, the effect of Ti content on the microstructure and properties of the laser-clad MPEA coatings was investigated. Detailed analyses of the coatings’ friction and wear performance, as well as cavitation erosion resistance, were conducted. Under a 20 N load for 30 min, increasing Ti content reduced the wear rate from 4.41 × 10^−5^ mm^3^/(N·m) to 3.79 × 10^−5^ mm^3^/(N·m), resulting in a 14.06% improvement in wear resistance. Cavitation erosion (CE) tests showed that after 20 h of ultrasonic exposure, the sample with x = 2.1 exhibited a cumulative mass loss of 4.92 mg, compared to 8.12 mg for the sample with x = 0.3. This represents a 39.4% reduction in cavitation erosion mass loss for the higher-Ti-content coating. In stark contrast, the Q235 substrate incurred a significantly higher mass loss of 86.78 mg under identical conditions. This signifies a dramatic enhancement in cavitation erosion resistance achieved by the high-Ti MPEA coating. These findings demonstrate a novel approach to enhancing the cavitation erosion resistance of MPEA claddings by optimizing Ti content, thereby broadening their potential application scope in harsh environments.

## 1. Introduction

Flow-passing components of hydraulic machinery often fail due to extensive cavitation damage. The continuous impingement of microjets induces surface plastic deformation, crack propagation, and material spalling, significantly shortening the service life of materials [1]. Consequently, fabricating cavitation-resistant composite coatings on these components through surface engineering technologies has become a research focus in material protection fields. Q235 steel is widely used as a structural material in practical engineering due to its excellent welding properties and low cost. However, due to its low hardness, poor cavitation resistance, and wear resistance, its service life is seriously limited. In order to solve this problem, the preparation of protective coating on the surface of the material is one of the effective methods of material protection at present [2,3,4]. Laser cladding technology has attracted significant attention in the field of surface modification and cavitation resistance due to its precise heat input control and high processing efficiency, particularly when combined with the exceptional mechanical properties of multi-principal element alloys [3,4]. Cavitation erosion (CE) refers to a phenomenon in hydraulic environments where complex fluid flow around high-speed moving components creates pressure variation zones. In low-pressure regions, vapor bubbles form and subsequently collapse violently, generating repeated localized pressure shock waves on component surfaces that cause severe material damage [5,6]. The cavitation resistance of the material mainly depends on its mechanical properties [7]. Of course, natural rivers usually contain suspended sediments, so the wear resistance of the material is also important to its cavitation resistance [8].

Multi-principal element alloys (MPEAs) depart from the conventional alloy design paradigm of single-element dominance, instead employing a multi-principal design strategy that transforms material performance from single-element dependence to multi-element synergy, thereby significantly enhancing alloy properties. The complex composition and multiscale structural interactions result in high mixing entropy that favors the formation of thermodynamically stable simple solid solutions, such as face-centered cubic (FCC) or body-centered cubic (BCC) structures, rather than intermetallic compounds or other complex phases [9,10]. This characteristic endows multi-principal element alloys (MPEAs) with tremendous application potential in extreme equipment for aerospace, hydroelectric power, and other demanding engineering fields [10,11,12].

The laser cladding multi-principal element alloy (MPEA) coating technology integrates the dual advantages of laser cladding processing and MPEA materials. As a frequently employed alloying element, titanium content variation significantly modifies the phase constitution of laser-clad coatings, consequently tailoring their performance. Liang et al. [13] successfully fabricated defect-free CoCrFeNiMoTi high-entropy alloy (HEA) coatings on Q235 steel using laser cladding technology, systematically investigating the effect of Ti content on microstructure and wear resistance. The results demonstrate that with increasing Ti content, the volume fraction of the BCC phase increases, while the FCC phase decreases correspondingly. The average coating hardness progressively rises, reaching approximately 6.5 times that of the Q235 substrate. Wear track analysis revealed that the CoCrFeNiMoTi coatings exhibit significantly superior wear resistance compared to the substrate. This enhancement is attributed to the synergistic interaction among the constituent phases (FCC, BCC, and NiTi), where mechanical property complementarity and microstructure optimization collectively improve wear performance. Yin et al. [14] fabricated AlCoCrCuFe alloy coatings on 304 stainless steel substrates via laser cladding technology and evaluated their cavitation erosion resistance. Experimental results revealed that the laser-clad specimens exhibited a dual-phase (BCC + FCC) structure, with the dendritic microstructure demonstrating enhanced cavitation erosion resistance. After 20 h of cavitation testing, the coating surface exhibited exposed BCC-phase dendritic structures due to the preferential removal of interdendritic regions, providing direct evidence that phase morphology governs cavitation erosion resistance.

Titanium inherently forms a stable passive film that provides effective protection in various corrosive media, thereby enhancing the cavitation erosion resistance of alloy coatings. Wang et al. [15] analyzed the cavitation and corrosion–cavitation synergistic behavior of a CoCrFeNiMnTix alloy coating, in which the samples with x = 1.0 showed the best anti-cavitation performance due to the lowest weight loss rate and the lowest cavitation rate. Zhao et al. [16] fabricated a CoCrFeNiMn alloy cladding layer on 304 stainless steel using coaxial powder feeding laser cladding technology. Experimental results revealed that following 10 h of cavitation erosion, the passive film on the coating demonstrated the lowest electrochemical current density. Additionally, the coating exhibited a significantly lower mean depth erosion rate compared to the 304 stainless steel. The superior cavitation erosion resistance performance of the cladding coating is attributed to its refined grain structure effectively resisting plastic deformation, while the surface passive film maintained high integrity and continuity throughout prolonged cavitation exposure. Furthermore, current research on the cavitation erosion resistance of laser-clad multi-principal element alloys remains limited, with no existing studies specifically addressing the cavitation erosion and wear resistance of Fe_66.5−x_Cr_24_Ni_8_Nb_1.5_Ti_x_ alloy coatings. This study systematically investigates the influence of Ti content on the wear resistance and cavitation erosion performance of laser-clad Fe_66.5−x_Cr_24_Ni_8_Nb_1.5_Ti_x_ multi-principal element alloy coatings. The primary objective is to address material degradation issues in hydraulic turbine components under harsh operating conditions, ultimately aiming to reduce material loss and extend service lifetime.

## 2. Materials and Experimental

### 2.1. Preparation of Raw Materials and Coatings

Q235 steel was chosen as the substrate with dimensions of 100 mm × 50 mm × 30 mm. The nominal chemical compositions of Fe_66.5−x_Cr_24_Ni_8_Nb_1.5_Ti_x_ alloy powders are listed in Table 1. Before laser cladding, the substrate was ground sequentially with 80–600 grit sandpaper, followed by alcohol cleaning and oven drying. Fe, Cr, Ni, Nb, and Ti powders (>99.9 wt% purity) were homogenously mixed using a SYH-5 three-dimensional motion powder mixer(Qunwei, Changzhou, China). The powder mixture was homogenized for 10 h to ensure optimal uniformity. The homogenized alloy powder mixture was loaded into the powder feeder for subsequent laser cladding processing using a coaxial powder feeding system (LASERLINE, LDF4000, Mülheim-Kärlich, Germany). The coating was deposited onto Q235 steel, using argon as the shielding gas during the laser cladding process. Through preliminary experiments, the melting parameters for controlling thickness and forming quality were determined as follows: the laser power was 3.2 kW, the scanning speed was 8 mm/s, the overlap rate was 50%, and the powder feeding speed was 40 g/min. Seven coating materials were designed, and two representative coatings were selected for detailed study after the study of their hardness. The corresponding titanium contents were 0.3% and 2.1%, which were marked as Ti0.3 and Ti2.1.

### 2.2. Performance Testing

The surface morphologies and elemental compositions of the coatings were analyzed using a scanning electron microscope (SEM; Zeiss SUPRA55, Oberkochen, Germany) equipped with an energy-dispersive spectrometer (EDS; Zeiss Merlin Compact, Oberkochen, Germany). The phase compositions of the coatings were characterized by X-ray diffraction (XRD; X’Pert PRO, Panalytical, Almelo, The Netherlands). Microhardness was measured using a Vickers microhardness tester (HXD-1000IMGCD, Taiming, Shanghai, China) with an indentation load of 1.96 N and a dwell time of 10 s. Ten indentations were made per sample. The reported microhardness values represent averages of these ten measurements, with the error indicated as the standard deviation. Using an ultrasonic vibrator for the vibration cleaning test in an alcohol solution (25 ± 2 °C) and after cleaning and drying, weight loss measurements were conducted. The large specimens coated with the material were cut into test-sized specimens using a molybdenum wire-cutting machine for microstructure characterization. The cut specimens were sequentially polished with 180#, 400#, 600#, 1000#, 1200#, and 2000# sandpaper, then polished with 3-micron and 0.05-micron polishing liquids. The samples were ultrasonically cleaned for 10 min and dried at 80 °C for 30 min.

The friction wear and cavitation resistance of the alloy coating were further studied with different Ti contents. The wear resistance of the coating was analyzed by friction and a wear tester (UMT Tribo Lab™, Bruker, Ettlingen, Germany). The wear tests were conducted using a 5 mm diameter Si_3_N_4_ ball as the counter material under the following conditions: the normal load was 20 N, the frequency was 3 Hz (reciprocating motion), the sliding distance was 10 mm (amplitude: ±5 mm), and the test duration was 30 min. Following wear testing, the worn surfaces were subjected to comprehensive microstructural and compositional analysis using scanning electron microscopy (SEM) coupled with energy-dispersive X-ray spectroscopy (EDS) to elucidate wear mechanisms and tribochemical interactions. The wear track morphology was quantitatively analyzed using a three-dimensional (3D) optical profilometer. The samples were polished to a mirror finish and then mounted in the cavitation erosion test rig (XO1200, 50 μm, Nanjing, China) for evaluation under the following conditions: a cavitation erosion test was carried out in deionized water at a temperature of 20 ± 2 °C for 20 h; then, the cavitation erosion performance of the coating was compared and analyzed.

## 3. Results and Discussion

### 3.1. Microscopic Morphology and Mechanical Properties

**Figure 1:**
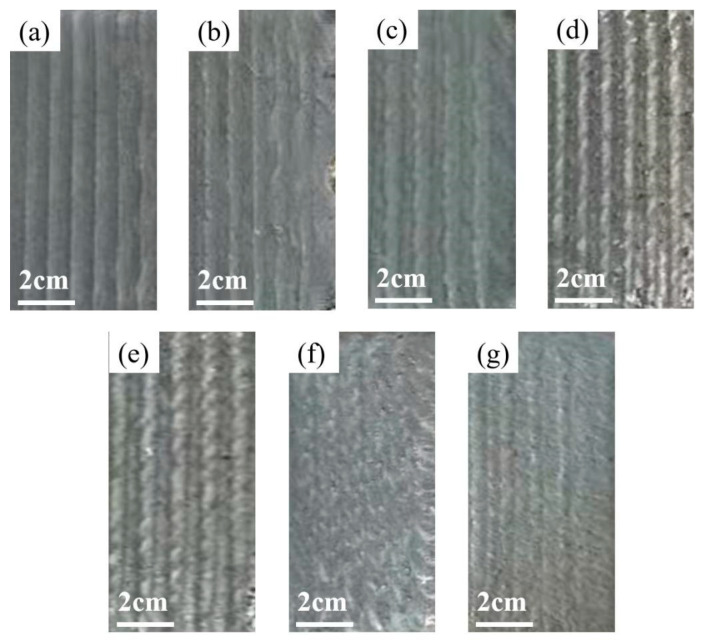
Macroscopic morphology of laser cladding of different Ti content alloy coatings: (**a**) Ti0.3, (**b**) Ti0.6, (**c**) Ti0.9, (**d**) Ti1.2, (**e**) Ti1.5, (**f**) Ti1.8, (**g**) Ti2.1.

Figure 1 displays the macroscopic surface morphology of laser-clad coatings with different Ti concentrations. Experimental observations revealed that coatings with x ≤ 2.1 maintained structural integrity without detectable cracks or porosity. However, at x > 2.1, distinct surface cracking became evident, likely attributable to the precipitation of hard brittle phases and elemental segregation induced by excessive Ti content, which facilitated crack initiation and propagation.

**Figure 2:**
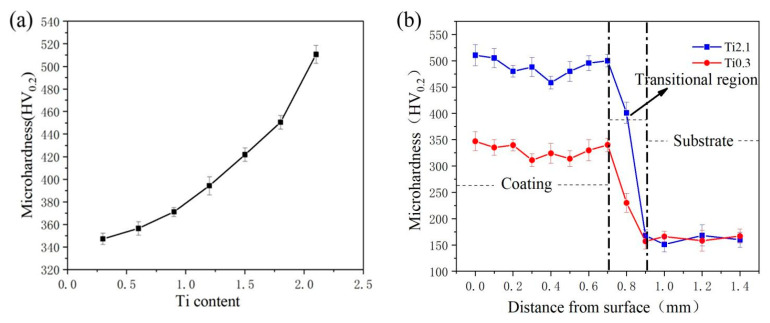
Hardness distribution of alloy coating: (**a**) surface hardness of Ti0.3-Ti2.1 samples, (**b**) hardness distribution across Ti0.3, Ti2.1 coatings, and base material.

Figure 2a presents the microhardness measurements of alloy coatings with varying Ti contents. Notably, all coatings demonstrate significantly higher microhardness values compared to the substrate material, with a clear trend of increasing hardness corresponding to a higher Ti content. The microhardness profiles of the Ti0.3 and Ti2.1 coatings are presented in Figure 2b. The surface hardness of the Ti2.1 coating (510 HV_0.2_) is significantly higher than that of the Ti0.3 coating (347 HV_0.2_). The microstructural analysis indicates that this mechanical behavior stems from Ti-content-dependent phase transformations; the Ti2.1 coating predominantly consisted of BCC phase structure, resulting from Ti-induced phase transformation during laser deposition. This microstructural evolution increased the hardness of the material.

**Figure 3:**
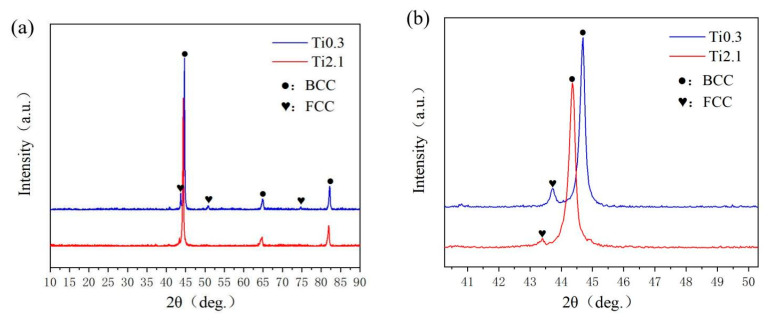
(**a**) XRD patterns of Fe_66.5−x_Cr_24_Ni_8_Nb_1.5_Ti_x_ alloy coatings; (**b**) partial enlarged drawing.

Figure 3a presents the XRD patterns of the samples. With increasing Ti content, the BCC phase gradually increases, demonstrating that an appropriate amount of Ti enhances material strength while excessive Ti suppresses FCC phase formation. The microstructure of the samples primarily consists of a dual-phase mixture of body-centered cubic (BCC) and face-centered cubic (FCC) structures. The Ti0.3 and Ti2.1 coatings predominantly consist of the BCC phase, and the Ti2.1 coating is harder than the Ti0.3 coating, resulting from a strengthening mechanism transition from solid solution strengthening to a mixed mechanism combining the solid solution and hard phase precipitation.

When the Ti content increases, the diffraction peak of the BCC phase is enhanced, while the FCC phase decreases. This indicates that the addition of excess Ti element promotes the formation of the BCC phase in the coating, thus reducing the proportion of the FCC phase. The precipitation of hard phases such as FeNi will inhibit the FCC phase and form BCC or intermetallic compounds instead, enhancing the surface hardness of the coatings. This phenomenon corroborates the observed higher microhardness of the Ti2.1 coating compared to Ti0.3. As shown in Figure 3b, the XRD magnified figure of Ti0.3 and Ti2.1 shows that the BCC peak tends to shift to the left. With the increase in Ti content, the lattice periodicity of the BCC phase is changed, and the proportion of Ti in the BCC phase increases. It may be that the lattice parameter is reduced through the strength of interatomic bonding, thus affecting the XRD peak position and causing the left shift [17].

Laser cladding technology, characterized by rapid cooling and heating, significantly increases the degree of supercooling and overheating. This results in a significant thermal expansion coefficient between the multi-component alloy coating and the substrate, leading to residual tensile stress and a higher likelihood of cracking [18,19]. However, by continuously optimizing process parameters and performing heat treatment before and after cladding, the formation of cracks can be greatly reduced. The microstructure of the upper, middle, and lower sections of the Ti0.3- and Ti2.1-coated samples is shown in Figure 3. The grain boundaries are clearly visible, with no obvious defects and good density. It is observed that as the Ti content increases, the grain boundaries become more pronounced. This is due to the gradual segregation of Ti elements into the grain boundaries, leading to their enrichment and the gradual segregation becoming more prominent.

**Figure 4:**
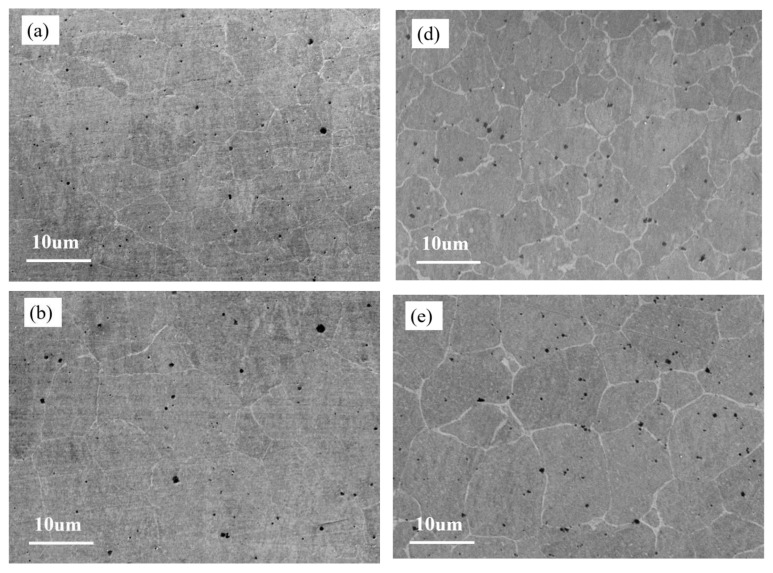

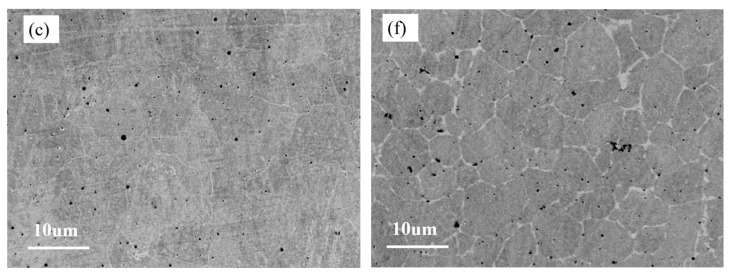
Microstructure of laser cladding alloy coating: (**a**) upper part of Ti0.3 coating, (**b**) middle part of Ti0.3 coating, (**c**) lower part of Ti0.3 coating, (**d**) upper part of Ti2.1 coating, (**e**) middle part of Ti2.1 coating, (**f**) lower part of Ti2.1 coating.

Comparing Figure 4a–c, it is evident that the grain diameter at the top of the coating is the smallest, followed by the bottom, with the middle having the largest grain diameter. This is because the top part cools the fastest, radiating heat to the air, which prevents the grains from growing sufficiently, resulting in the smallest grain diameter at the top. The bottom part cools through heat conduction to the matrix material. However, due to the matrix material being preheated to 473.5 K, although the cooling is relatively fast, the preheating affects the growth time of the grains, making them slightly longer than those at the top, and thus the grain diameter at the bottom is slightly larger. The middle part, with slower cooling, has more time for grain growth, making the grain diameter significantly larger. Similarly, comparing Figure 4d–f yields the same conclusion. This also explains why the microhardness of the coating is higher at the top and bottom compared to the middle and why the hardness at the top is slightly higher than that at the bottom. As can be seen from Figure 1, the grain diameter of the T2.1 coating is smaller than that of T0.3, for the following reasons. Firstly, the supercooling of the melt is enhanced by the addition of Ti. The melting point of Ti (1668 °C) is significantly higher than those of Fe and Ni. When Ti is introduced into the melt pool, it elevates the liquidus temperature of the alloy system, thereby increasing the undercooling degree. This higher undercooling promotes a substantial rise in the nucleation rate, leading to refined grain structures. Secondly, the addition of Ti causes lattice distortion. The atomic radius of Ti (147 pm) is markedly larger than that of Fe (126 pm) and Ni (124 pm). The incorporation of Ti induces severe lattice distortion within the solid solution, which increases the system energy. This energy surge accelerates grain boundary formation and enhances grain refinement. Thirdly, Ti addition facilitates the formation of the body-centered cubic (BCC) phase. As the Ti content increases, the phase structure evolves from a mixed FCC + BCC phase to a predominantly BCC-dominated structure. The BCC phase exhibits a higher nucleation energy barrier compared to the face-centered cubic (FCC) phase, effectively suppressing grain growth and refining the microstructure. These results also explain why the microhardness of the T2.1 coating is significantly higher than that of the T0.3 coating.

In order to clarify the composition of the coating after laser cladding, EDS analysis was carried out on the surface composition of the T0.3 and T2.1 coatings after grinding and polishing. The results are shown in Table 2. As can be seen from Table 2, the surface composition of coating after grinding and polishing is close to the design composition. This is because the surface of coatings are far away from the matrix and are not significantly diluted in the laser cladding process, so they can maintain a basic consistency with the design composition.

**Figure 5:**
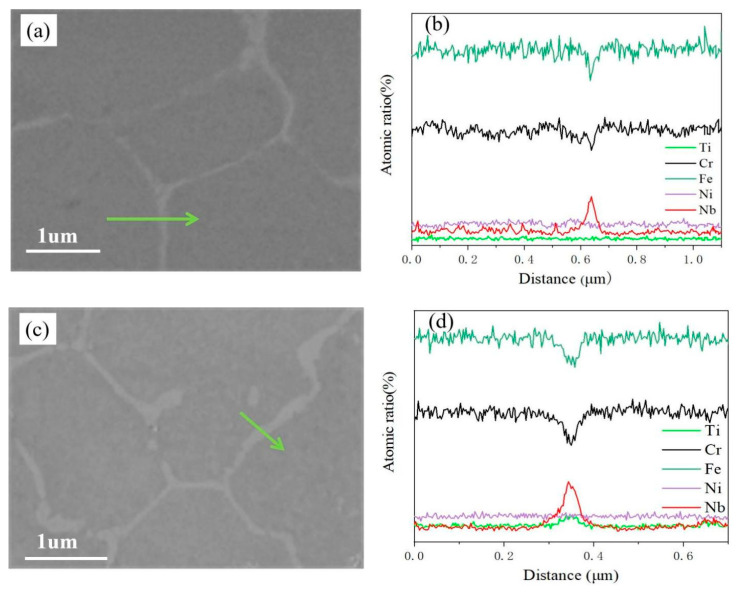
Distribution of elements at and around the coating grain boundary: (**a**,**c**) the microstructure of the scan position, (**b**) the distribution of elements around the Ti0.3 grain boundary, (**d**) the distribution of elements around the Ti2.1 grain boundary.

To analyze the elemental distribution at grain boundaries and their surrounding regions, line scanning was performed across the grain boundaries *(The green arrow indicates the direction of the line scan),* as shown in Figure 5. Figure 5a,b reveal that Nb is enriched at the grain boundaries of the coating, while Fe and Cr are relatively depleted. With increasing Ti content, as shown in Figure 5c,d, Ti gradually diffuses toward the grain boundaries, and the concentration of Nb at the grain boundaries also slightly increases. This is because titanium is typically more chemically active than niobium and tends to preferentially occupy vacancy sites under high-temperature conditions, thereby altering the local chemical environment within the material. In addition, the atomic radius and chemical properties of Nb and Ti are similar, and the increase in Ti will reduce the solubility of Nb in the matrix and promote the segregation of Nb to grain boundaries [19].

### 3.2. Friction Wear Performance

**Figure 6:**
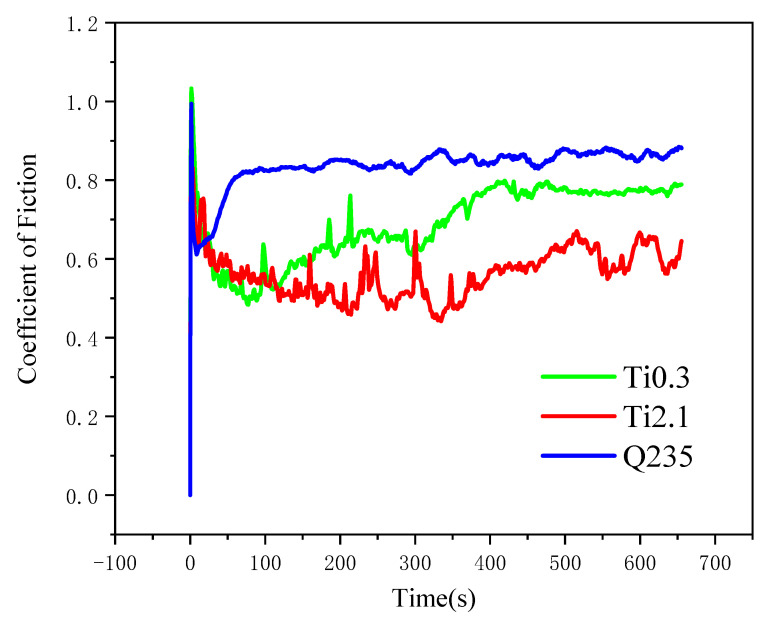
Friction wear curve of laser cladding alloy coating.

Figure 6 illustrates the curves of the friction coefficients of alloy coatings with varying Ti contents as they change over time. The friction coefficient of Ti0.3 is notably higher than that of Ti2.1. At the start of the wear test, the friction coefficient initially decreases due to the formation of a friction film between the sample surface and the friction pair, which reduces shear resistance. As time progresses, the contact area between the friction pair and the sample increases, and the coefficient of friction gradually increases and then tends to stabilize.

**Figure 7:**
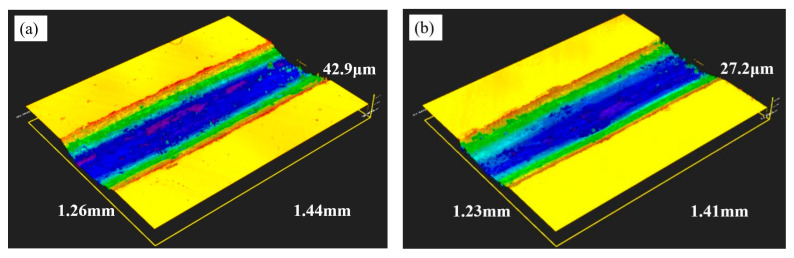

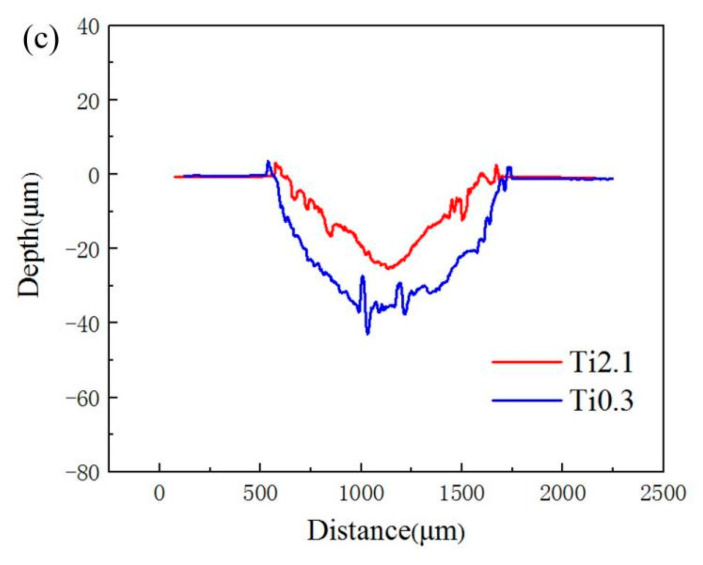
Three-dimensional surface topography and two-dimensional cross-section profile of alloy coatings after wear under 20 N load: (**a**) Ti0.3, (**b**) Ti2.1, (**c**) 2d cross-section profile of wear marks.

Based on the relationship between the material friction coefficient and wear resistance, the friction coefficient and weight loss of Ti2.1 are the lowest. This may be because an increase in Ti content can lead to grain boundary pinning and the formation of new compounds during rapid cooling, enhancing wear resistance [20]. Additionally, the introduction of Ti elements leads to a gradual shift towards a single BCC phase structure, improving hardness and wear resistance. The two-dimensional and three-dimensional wear morphology of the surfaces of the two coatings at room temperature are shown in Figure 7 *(The change in color indicates depth differences).* The coating surface developed unevenly deep grooves along the direction of friction. The Ti2.1 alloy coating exhibited the best wear resistance, with the smallest wear depth over the same distance range. The formula for calculating the wear rate of the coating is W = V_loss/_(F_N_·H), where W represents the wear rate, V_loss_ is the volume of wear, F_N_ is the load, and H is the total sliding distance. Table 3 shows the wear rate of the Ti0.3 and Ti2.1 coatings as well as Q235 under a load of 20 N.

**Figure 8:**
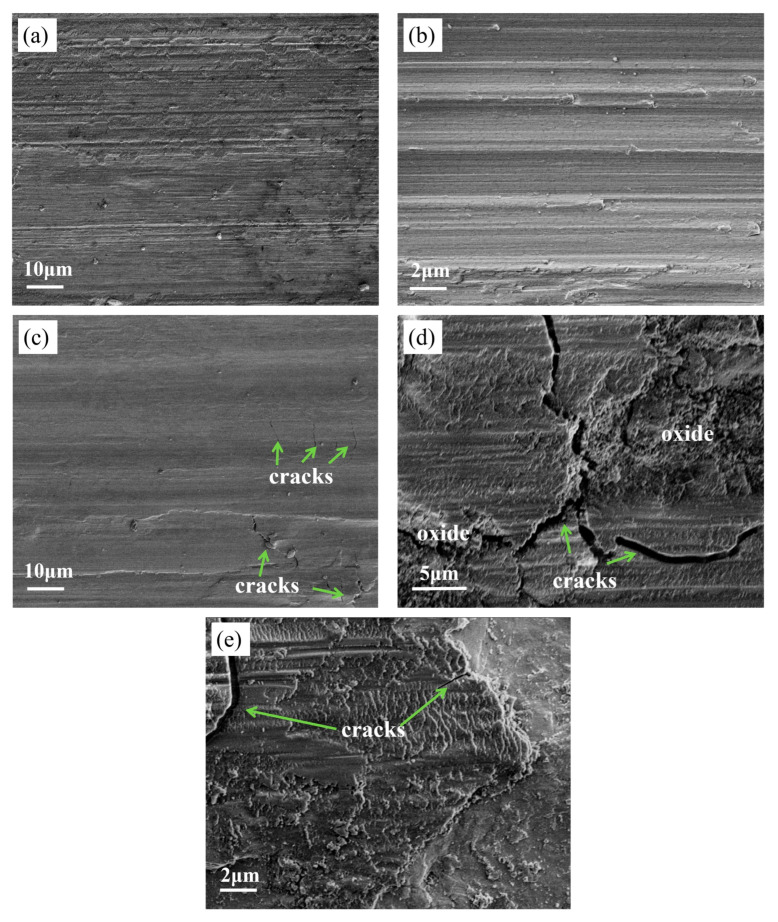
(**a**) Microscopic morphology of Ti0.3 coating after wear; (**b**) local magnified micrographs of Ti0.3 coating after wear; (**c**) microscopic morphology of Ti2.1 after wear; (**d**,**e**) local magnified micrographs of Ti2.1 coating after wear.

**Figure 9:**
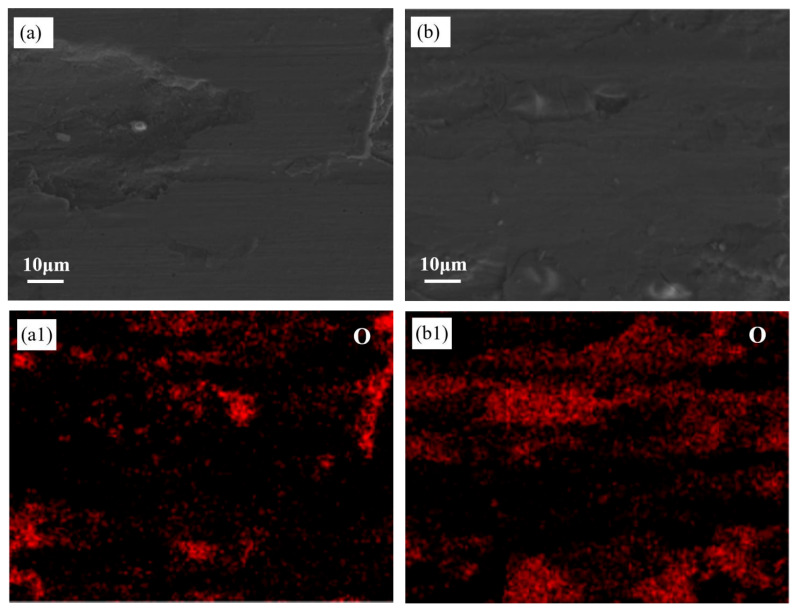

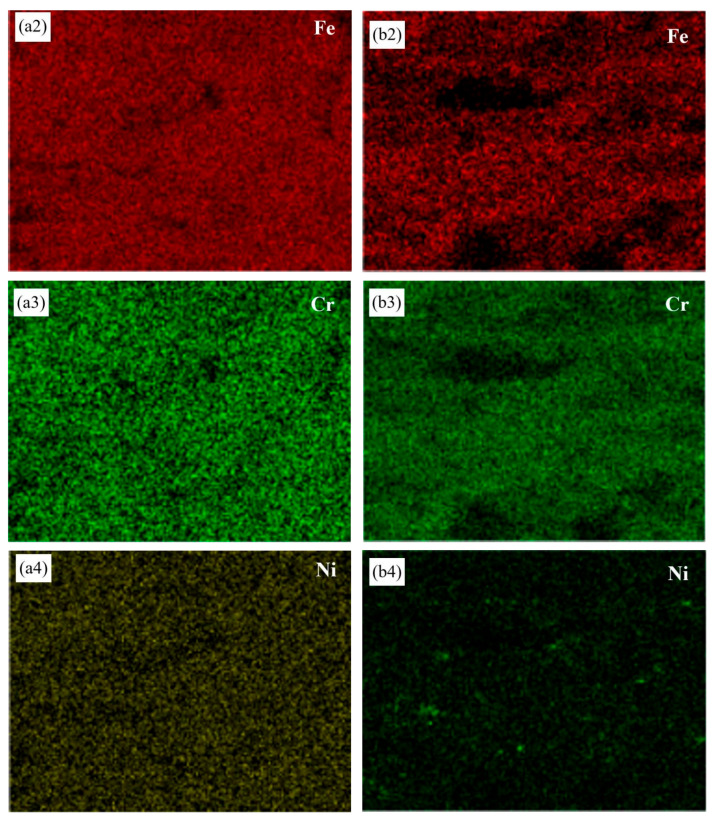

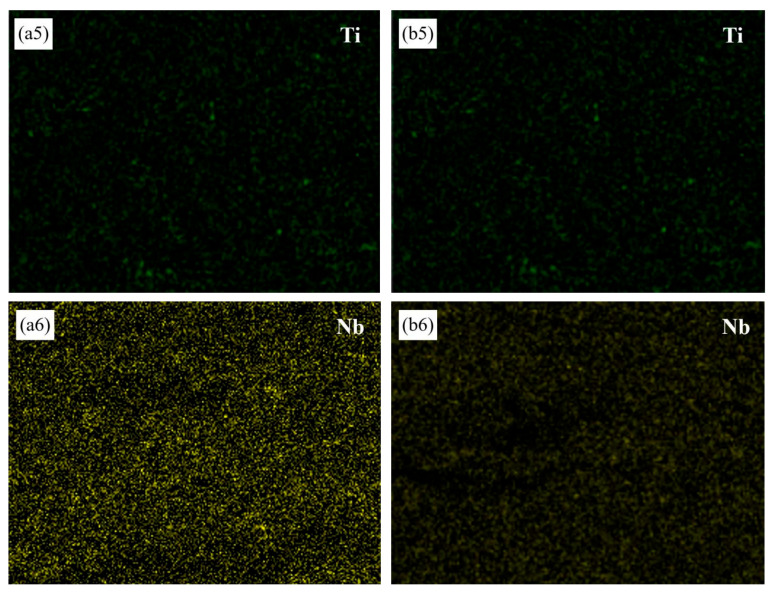
Surface composition distribution after wear of laser cladding alloy coating under 20 N load: (**a**,**a1**–**a6**) Ti0.3, (**b**,**b1**–**b6**) Ti2.1.

Figure 8 shows the microstructure after wear when the load is 20 N. Figure 8a,c show the microstructure of the Ti0.3 and Ti2.1 samples after wear. As can be seen from Figure 8a, there is no obvious crack on the surface of the T0.3 coating after wear, and micro-cutting occurs on the surface, showing a ploughing groove morphology, indicating that the wear mechanism of the T0.3 coating belongs to the typical abrasive wear. As can be seen from Figure 8b, over time, the coating peels off more severely, and more abrasive particles are produced. The microstructure of Ti2.1 after wear was analyzed, and it was found that some microcracks and spalling marks caused by brittle fracture appeared. The higher hardness of Ti2.1 leads to stress concentration and toughness deficiencies during wear, resulting in cracks. Additionally, Figure 8d reveals that the worn T2.1 coating surface displays a porous oxidized microstructure, indicating oxidation-induced degradation. This observation confirms the occurrence of oxidative wear on the T2.1 coating. Such oxidative wear is further substantiated by the EDS mapping data presented in Figure 9. Notably, visible cracks observed on the worn surface in Figure 8e demonstrate fatigue wear resulting from cyclic contact stresses, and the morphology of layered exfoliation is presented. Thus, the T2.1 coating exhibits a composite wear mechanism dominated by fatigue wear accompanied by significant oxidative wear.

Figure 9 illustrates the elemental distribution after wear at a 20 N load on both coatings. It can be observed that the distribution of elements in the coating is relatively uniform, and oxygen elements exist in all of them and are locally covered on the surface of the grinding mark. This is attributed to the heat generated in the reciprocating friction process, and the oxidation layer formed during wear is attached to the worn surface, resulting in oxidative wear in some areas. This conclusion can also be confirmed by the data reflected in SEM images in Figure 8c,d. Comparing Figure 9(a1,b1), it is evident that the oxidative wear is more pronounced in the T2.1 coating than in the T0.3 coating. This phenomenon is primarily attributable to the higher content of the BCC phase in the T2.1 coating microstructure. The enhanced BCC phase fraction strengthened the coating hardness, thereby rendering the coating more resistant to being plowed through during wear testing. Consequently, this led to localized temperature elevation, which promoted thermal oxidation.

### 3.3. Cavitation Erosion Performance

**Figure 10:**
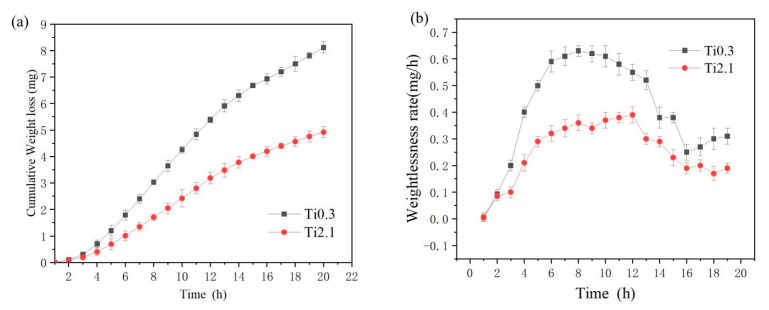
Weight loss curve of laser cladding alloy coating under cavitation erosion with time: (**a**) weight loss at different times, (**b**) weight loss rate at different times.

The weight loss of the Ti0.3 and Ti2.1 samples under cavitation with time is shown in Figure 10. As shown in Figure 10a, both coatings exhibited negligible weight loss within the initial 1.5 h of cavitation time. However, with prolonged exposure, the weight loss of both coatings progressively increased. Figure 10b reveals that after undergoing the incubation period of cavitation, the two coatings initiated weight loss, with their weight loss rates demonstrating a trend of rapidly increasing values followed by gradually slowing growth, eventually declining and tending toward stabilization. There was almost no mass loss of the two samples in the first 1.5 h, indicating that the alloy coating was in the initial incubation stage, which was characterized by the material’s ability to effectively buffer the impact of external microjet flow through its own plastic deformation capacity, thus delaying the surface damage process. As can be seen from Figure 10a and Table 4, the Ti2.1 sample shows the best cavitation resistance, with only 4.92 mg mass loss after 20 h of cavitation. The weight loss of Ti0.3 cavitation after 20 h was 8.12 mg, while the weight loss of Q235 after 20 h of cavitation was 86.78 mg, which greatly improves the cavitation resistance of turbine materials. XRD analysis indicates that when the FCC phase and BCC phase coexist in the coating, the FCC phase tends to undergo plastic deformation through dislocation slip and deformation twinning. The BCC structure, with its higher lattice distortion energy and grain boundary density, effectively hinders dislocation movement [16]. As the content of BCC-stabilizing elements like Ti increases, the alloy transitions from a state where the FCC and BCC phases coexist to a single BCC phase, enhancing the coating’s hardness. This makes the high-hardness BCC phase more resistant to microjet-induced plastic deformation during cavitation impact, thereby reducing material spalling.

**Figure 11:**
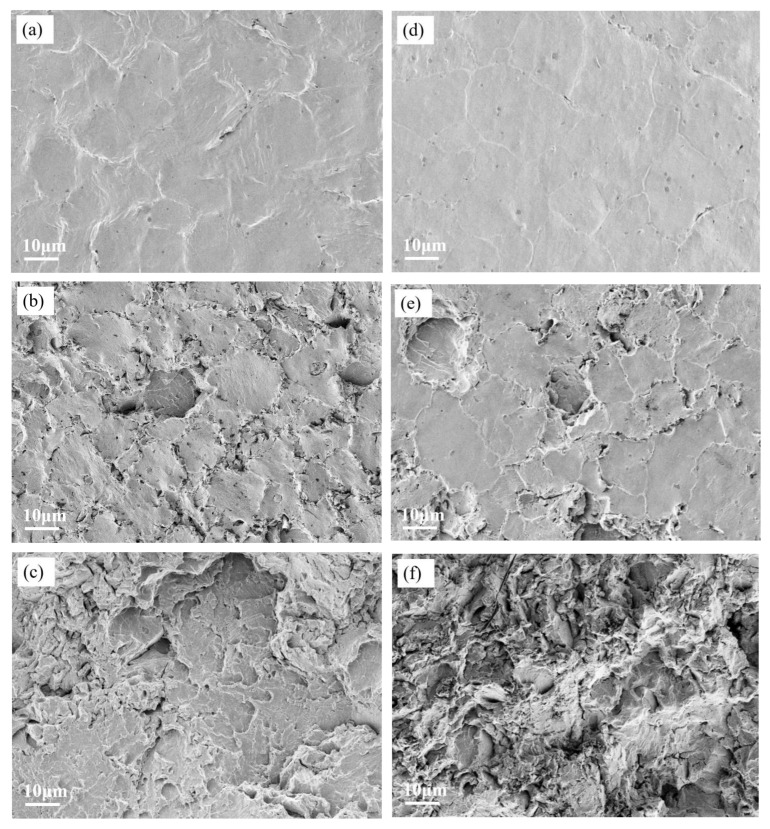
The surface microstructure of laser cladding alloy coatings after cavitation erosion: (**a**) Ti0.3 cavitation after 1 h, (**b**) Ti0.3 cavitation after 4 h, (**c**) Ti0.3 cavitation after 20 h, (**d**) Ti2.1 cavitation after 1 h, (**e**) Ti2.1 cavitation after 4 h, (**f**) Ti2.1 cavitation after 20 h.

Figure 11 presents the post-cavitation microstructures of the coatings. From the figure, the cavitation erosion (CE) process can be observed. After 1.5 h of CE exposure, both coatings exhibited plastic deformation without surface spalling, indicating that both were in the CE incubation period. Among them, the T0.3 coating showed significantly more pronounced plastic deformation than the T2.1 coating. After 4 h of CE, spalling occurred on both coatings, suggesting that they entered the CE acceleration stage. Notably, the grain boundaries of T0.3 experienced more severe spalling compared to T2.1. This also reveals that the grain boundary strength of T2.1 is higher than that of T0.3. After 20 h of CE, both coatings underwent complete surface spalling. The T0.3 coating surface displayed quasi-cleavage fracture characteristics—a mixed-mode fracture morphology comprising cleavage facets, dimples, and tear ridges. The T2.1 coating also exhibited mixed-mode fracture features, with transgranular fracture, dimple rupture, and fatigue fracture accompanied by secondary cracks coexisting.

Through systematic investigation, the material peeling caused by plastic deformation is more serious in the Ti0.3 coating. This phenomenon is attributed to the combined action of high-pressure shockwaves and microjet impacts generated by collapsing bubbles in the cavitation environment. The observed severe spalling and mass loss in Ti0.3 after 20 h of cavitation exposure result from stress concentration-driven pore propagation. After 20 h of cavitation exposure, the Ti2.1 coating surface developed localized irregular pores and numerous dimples.

## 4. Conclusions

(1) Compared with the Ti0.3 coating, the structure of the Ti2.1 coating changed to a single BCC phase. This microstructural evolution led to an increase in coating hardness from 347 HV_0.2_ to 510 HV_0.2_.
(2) The increase in alloy coating wear resistance was driven by higher Ti content. This was demonstrated by dry sliding wear tests under a 20 N load for 30 min: the wear rate decreased from 4.41 × 10^−5^ mm^3^/(N·m) (x = 0.3) to 3.79 × 10^−5^ mm^3^/(N·m) (x = 2.1). The wear resistance of the coating with x = 2.1 showed a 24.2% improvement compared to the base material (Q235 steel).
(3) Compared with the Ti0.3 coating, the coating corresponding to x = 2.1 exhibited better performance, sustaining a mass loss of only 4.92 mg after 20 h of ultrasonic cavitation testing. In stark contrast, the Q235 substrate incurred a significantly higher mass loss of 86.78 mg under identical conditions. This signifies a dramatic enhancement in cavitation erosion resistance achieved by the high-Ti MPEA coating.

## Figures and Tables

**Table 1:** The nominal chemical compositions of Fe_66.5−x_Cr_24_Ni_8_Nb_1.5_Ti_x_ alloy powders.

Sample/Chemical Composition (at.%)	Fe	Cr	Ni	Nb	Ti
Ti0.3	66.2	24	8	1.5	0.3
Ti0.6	65.9	24	8	1.5	0.6
Ti0.9	65.6	24	8	1.5	0.9
Ti1.2	65.3	24	8	1.5	1.2
Ti1.5	65.0	24	8	1.5	1.5
Ti1.8	64.7	24	8	1.5	1.8
Ti2.1	64.4	24	8	1.5	2.1

**Table 2:** The chemical compositions of Ti0.3 and Ti2.1 coatings by EDS.

Sample/Chemical Composition(at.%)	Fe	Cr	Ni	Nb	Ti
Ti0.3	66.08	24.34	7.81	1.48	0.29
Ti2.1	63.76	24.51	8.03	1.53	2.08

**Table 3:** Wear rate analysis under varying loads.

Sample/Load (N)	20 N
Ti0.3	4.41 × 10^−5^ mm^3^/N·m
Ti2.1	3.79 × 10^−5^ mm^3^/N·m
Q235	5.0 × 10^−5^ mm^3^/N·m

**Table 4:** The weight loss of cavitation after 20 h.

Sample	Weight Loss (mg)
Ti0.3	8.12
Ti2.1	4.92
Q235	86.78

## Data Availability

The original contributions presented in this study are included in the article. Further inquiries can be directed to the corresponding authors.
